# The matrix domain contributes to the nucleic acid chaperone activity of HIV-2 Gag

**DOI:** 10.1186/s12977-016-0245-1

**Published:** 2016-03-17

**Authors:** Katarzyna Pachulska-Wieczorek, Leszek Błaszczyk, Marcin Biesiada, Ryszard W. Adamiak, Katarzyna J. Purzycka

**Affiliations:** Institute of Bioorganic Chemistry, Polish Academy of Sciences, Noskowskiego 12/14, 61-704 Poznan, Poland; Institute of Computing Science, Poznan University of Technology, Piotrowo 2, 60-965 Poznan, Poland

**Keywords:** HIV-2, Gag, Matrix, Nucleocapsid, Nucleic acid chaperone, tRNA^Lys3^ annealing, RNA binding proteins, RNA dimerization, RNA structure

## Abstract

**Background:**

The Gag polyprotein is a multifunctional regulator of retroviral replication and major structural component of immature virions. The nucleic acid chaperone (NAC) activity is considered necessary to retroviral Gag functions, but so far, NAC activity has only been confirmed for HIV-1 and RSV Gag polyproteins. The nucleocapsid (NC) domain of Gag is proposed to be crucial for interactions with nucleic acids and NAC activity. The major function of matrix (MA) domain is targeting and binding of Gag to the plasma membrane but MA can also interact with RNA and influence NAC activity of Gag. Here, we characterize RNA binding properties and NAC activity of HIV-2 MA and Gag, lacking p6 domain (GagΔp6) and discuss potential contribution of NC and MA domains to HIV-2 GagΔp6 functions and interactions with RNA.

**Results:**

We found that HIV-2 GagΔp6 is a robust nucleic acid chaperone. HIV-2 MA protein promotes nucleic acids aggregation and tRNA^Lys3^ annealing in vitro. The NAC activity of HIV-2 NC is affected by salt which is in contrast to HIV-2 GagΔp6 and MA. At a physiological NaCl concentration the tRNA^Lys3^ annealing activity of HIV-2 GagΔp6 or MA is higher than HIV-2 NC. The HIV-2 NC and GagΔp6 show strong binding to the packaging signal (Ψ) of HIV-2 RNA and preference for the purine-rich sequences, while MA protein binds mainly to G residues without favouring Ψ RNA. Moreover, HIV-2 GagΔp6 and NC promote HIV-2 RNA dimerization while our data do not support MA domain participation in this process in vitro.

**Conclusions:**

We present that contrary to HIV-1 MA, HIV-2 MA displays NAC activity and we propose that MA domain may enhance the activity of HIV-2 GagΔp6. The role of the MA domain in the NAC activity of Gag may differ significantly between HIV-1 and HIV-2. The HIV-2 NC and MA interactions with RNA are not equivalent. Even though both NC and MA can facilitate tRNA^Lys3^ annealing, MA does not participate in RNA dimerization in vitro. Our data on HIV-2 indicate that the role of the MA domain in the NAC activity of Gag differs not only between, but also within, retroviral genera.

**Electronic supplementary material:**

The online version of this article (doi:10.1186/s12977-016-0245-1) contains supplementary material, which is available to authorized users.

## Background

The retroviral Gag polyprotein is not only a major structural component of immature virions, but also acts as multifunctional regulator of virus replication [1–4]. The Gag polyproteins of human immunodeficiency viruses type 1 and 2 (HIV-1 and HIV-2) share 50 % sequence homology and each contains the N-terminal matrix (MA) domain, which is responsible for Gag targeting and binding to the plasma membrane [5, 6], the capsid domain (CA), which facilitates Gag multimerization [4, 7], and the nucleocapsid (NC) domain that interacts with viral and cellular nucleic acids (NA) [2, 3, 8]. Gag also contains two spacer regions (SP1, SP2) and the p6 domain located at the C-terminus, which is necessary for virus release from the infected cell [9]. Before or shortly after virion release, the Gag polyprotein is cleaved in a highly ordered manner by viral protease into freestanding MA, CA, and NC proteins [1, 10]. In addition to the structural function in mature virions [4], the NC protein plays an important role in the facilitation of nucleic acid strand transfers during reverse transcription [11]. Mature MA remains bound to the viral membrane [4], but it was proposed that a fraction of HIV-1 MA also enters newly infected cells, associates with the pre-integration complex (PIC), and affects proviral DNA circularization and integration [12, 13].

At a late stage in the HIV replication cycle, Gag may be responsible for the annealing of tRNA^Lys3^ to an 18-nt primer binding sequence (PBS) localized in the 5′UTR of the viral RNA, where tRNA^Lys3^ primes reverse transcription [2, 14]. The NAC activity is considered necessary to anneal tRNA^Lys3^ but, so far, it has only been confirmed for HIV-1 and Rous sarcoma virus (RSV) Gag proteins in vitro [15–18]. Chaperone proteins can facilitate folding and formation of the most thermodynamically favoured structures of nucleic acids [19]. The NAC activity of HIV-1 Gag has been shown to depend on the NC domain, which is required for tRNA^Lys3^/viral RNA complex formation [14, 16, 17], whereas the MA domain can inhibit this process via RNA binding [17]. Numerous lines of evidence support the nucleic-acid-binding properties of retroviral MA [15, 20–27], but significant differences in the role of MA domain in the overall NAC activity of Gag were observed between retroviral genera. In contrast to HIV-1, the chaperone activity of alpharetrovirus RSV Gag is independent of the MA domain [15]. Moreover, HIV-1 and RSV NC display robust chaperone activity [15–17, 28], whereas MA proteins from both viruses do not promote annealing of primer tRNA in vitro [15–17]. The RNA-binding properties and chaperone activity of HIV-2 Gag have not been studied and the contribution of NC and MA domains remains undefined. We recently reported that HIV-2 NC is an effective NAC, but its activity is limited by the structural stability of the nucleic acid molecule to a much greater degree than that of HIV-1 NC [29].

As a nucleic acid chaperone, Gag binds NA non-specifically [2, 3], but is also engaged in highly specific recognition of *cis*-acting dimerization and packaging (Ψ) signals within the 5′UTR of the viral genomic RNA [30]. Although HIV-1 Gag binds GU-rich sequences in the cytoplasm, its binding specificity changes to A-rich RNA motifs during virion assembly [31]. The HIV-2 Gag-binding sites within the viral RNA remain uncharacterized and only limited information on HIV-2 NC binding to isolated domains of HIV-2 5′UTR in vitro is available [32, 33]. Similar packaging mechanisms are suggested for both viruses [34], but HIV-1 Gag is able to package HIV-2 RNA, whereas HIV-2 Gag cannot package HIV-1 RNA [35, 36]. The NC domains of HIV-1 and HIV-2 uncleaved Gag polyproteins are proposed to be crucial for the selection, dimerization, and packaging of viral RNA [8, 36–39]. In contrast, both NC and MA domains play direct roles in viral RNA packaging in deltaretroviruses [bovine leukaemia virus (BLV) and human T cell leukaemia virus type 2 (HTLV-2)] [22, 25, 26]. Interestingly, participation of the MA domain of HIV-1 Gag in these steps of viral replication was also suggested [23, 40, 41]. Moreover, an intriguing link between a mutation in the MA domain of HIV-2 Gag and viral RNA dimerization has been recently shown [42].

The three-dimensional structure of the entire HIV-1 and HIV-2 Gag is unknown, but the structures of their freestanding NC and MA have been presented [32, 43–46]. Moreover, the structure of the non-myristoylated HIV-1 Gag fragment (MA-CA-SP1-NC) was recently resolved by NMR spectroscopy [27]. The mature NC proteins of HIV-1 and HIV-2 are small basic proteins, containing two zinc finger domains (ZFs). The ZFs are proposed to be crucial for specific interactions of NC with nucleic acids, whereas basic residues from the disordered N-terminus play a role in non-specific interactions and NAC activity [8, 11, 29, 47]. Despite the limited sequence similarity between HIV-1 MA and HIV-2 MA, both proteins are composed of six α-helices and three β-sheet elements, and are myristoylated at the N-terminus [43, 44, 46]. The myristyl group and amino acid residues of HIV-1 and HIV-2 MA are engaged in PM binding [5, 43]. Importantly, some of those residues are located within the highly basic region (HBR) at the N-terminus of MA, which is proposed to be important for interactions with RNA in HIV-1 [21, 48–50]. RNA binding to the MA domain ensures the specificity of HIV-1 Gag interactions with PM phospholipids [6, 21, 48, 51]. Whether the MA domain of HIV-2 Gag is involved in RNA binding is not known.

Within this work we characterized the RNA-binding properties and nucleic acid chaperone activity of recombinant HIV-2 GagΔp6, NC and MA proteins. We identified binding sites of HIV-2 GagΔp6 and isolated NC and MA domains within the 5′UTR of HIV-2 RNA. Both HIV-2 NC and GagΔp6 show strong binding to the packaging signal and preference for the purine-rich sequences, while HIV-2 MA binds mainly to G residues without favouring Ψ RNA. Moreover, HIV-2 NC promotes HIV-2 RNA dimerization while this process is not supported by HIV-2 MA, suggesting that MA domain is dispensable for HIV-2 GagΔp6-promoted dimerization in vitro. We found that HIV-2 GagΔp6 is a robust nucleic acid chaperone and we propose that both NC and MA domains contribute to nucleic acids aggregation and tRNA^Lys3^ annealing in vitro. The NAC activity of HIV-2 NC is affected by salt in contrast to that of HIV-2 GagΔp6 and MA.

## Results

### Nucleic acid-binding properties of HIV-2 GagΔp6, MA, and NC

**Fig. 1:**
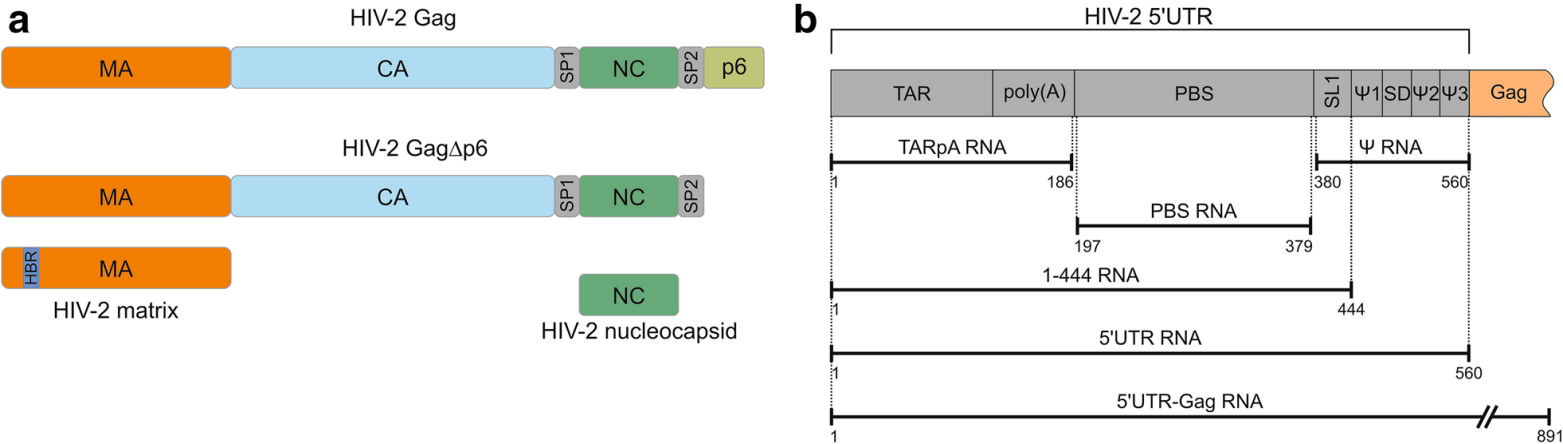
Schematic representation of HIV-2 proteins (**a**) and RNAs (**b**) used in this study

Interactions of HIV-1 Gag polyprotein as well as NC and MA proteins with nucleic acids are documented (reviewed in Ref. [3]). However, little information, and limited to freestanding NC [32, 33], is available for HIV-2. Therefore, the first step of our study was to characterize the binding of non-myristoylated HIV-2 Gag polyprotein lacking the p6 domain (referred to as GagΔp6) and freestanding HIV-2 NC and MA proteins to RNA. We performed salt-dependent filter-binding assays to examine interactions with three RNA molecules that represent functional domains of 5′UTR of HIV-2 genomic RNA: TARpA, PBS, and Ψ (Fig. 1) [33, 52, 53].

At physiological salt concentration (150 mM NaCl), the highest binding affinity was observed for Ψ RNA and HIV-2 GagΔp6 or NC (15 and 71 nM, respectively) (Table 1). However, GagΔp6 binding was salt-independent up to 250 mM NaCl, while NC affinity to Ψ RNA decreased nine-fold within a 100–250 mM NaCl range. At physiological salt concentration GagΔp6 displayed slightly lower affinity to PBS and TARpA than to Ψ RNA. A similar but more apparent trend was observed for HIV-2 NC protein, whereas HIV-2 MA bound PBS, TARpA and Ψ RNA with comparable affinities. Both HIV-2 NC and MA bound non-Ψ RNA molecules with similar affinities, but MA binding to Ψ RNA was significantly weaker than that of NC. Contrary to HIV-2 NC, GagΔp6 and MA consistently showed resistance to increasing salt within a 100–250 mM range, independently of the RNA substrate used. Importantly, even at 500 mM NaCl, HIV-2 GagΔp6 remained tightly bound to Ψ (Kd ≈ 125 nM) and PBS (Kd ≈ 314 nM) RNAs and only GagΔp6/TARpA interaction was reduced (Kd ≈ 965 nM). Both HIV-2 NC and MA proteins showed significantly lower affinity to RNA at 500 mM NaCl and only NC/Ψ RNA interaction was somewhat tighter.

**Table 1:** Calculated dissociation constants for HIV-2 NC, MA, and GagΔp6 complexes with selected RNA

NaCl (mM)	Kd (nM)		
	NC	MA	GagΔp6
TARpA RNA			
50	207 ± 9	431 ± 88	49 ± 5
100	285 ± 21	417 ± 74	52 ± 5
150	385 ± 16	430 ± 46	53 ± 5
200	541 ± 6	538 ± 68	65 ± 4
250	679 ± 16	593 ± 76	97 ± 7
500	1432 ± 22	2377 ± 209	965 ± 83
PBS RNA			
50	36 ± 3	335 ± 28	31 ± 1
100	117 ± 6	289 ± 4	31 ± 3
150	196 ± 9	290 ± 5	30 ± 2
200	277 ± 26	340 ± 25	31 ± 2
250	392 ± 37	382 ± 25	41 ± 3
500	2036 ± 212	2072 ± 98	314 ± 29
Ψ RNA			
50	7 ± 2	413 ± 52	27 ± 1
100	25 ± 1	300 ± 41	17 ± 1
150	71 ± 1	312 ± 29	15 ± 1
200	135 ± 4	357 ± 21	24 ± 2
250	240 ± 10	374 ± 19	26 ± 3
500	979 ± 26	1514 ± 210	125 ± 5

**Fig. 2:**
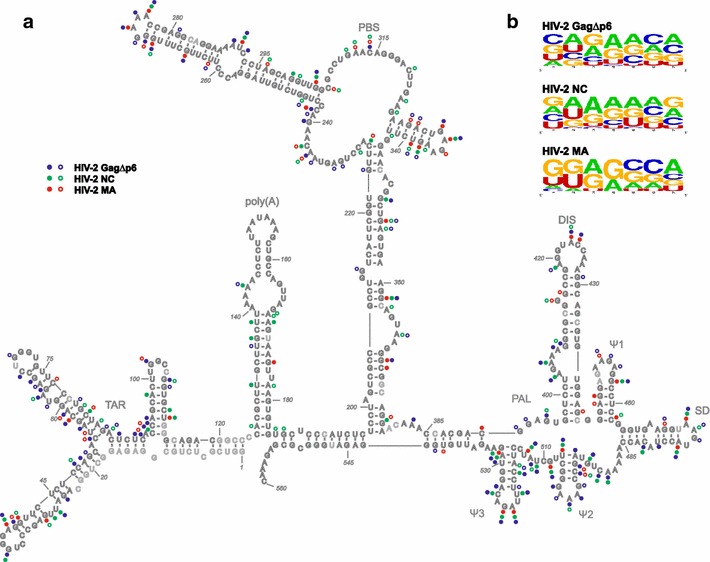
Protein binding to HIV-2 5′UTR. **a** Secondary structure model of +1–560 nt region of HIV-2 RNA [33] with the positions protected from hydroxyl radical cleavage in the presence of the HIV-2 GagΔp6, NC, and MA proteins marked. The level of protection increases with protein concentration. *Closed circles* indicate residues protected starting at lowest protein concentration; *open circles* indicate residues protected starting at intermediate protein concentration. *Light grey* annotates lack of data. **b** Sequences within binding sites and their vicinities were compared using http://weblogo.berkeley.edu/ [68]

Hydroxyl radical (HR) footprinting was used to further characterize HIV-2 GagΔp6, NC, and MA interactions with HIV-2 5′UTR. Overall reactivity profiles for RNA in the presence or absence of HIV-2 GagΔp6, NC, or MA were compared to reveal RNA sequences protected when proteins were present. At the highest protein concentration, almost the entire HIV-2 leader RNA was protected from HR cleavage for all proteins studied. The strongest decreases in HR cleavage were observed in the presence of HIV-2 GagΔp6 and NC proteins, while lesser effects were observed at the same concentration of HIV-2 MA, which supports the RNA-binding analysis (Table 1). A strong decrease in susceptibility to HR was noted for all studied proteins within TAR, PBS, and domains within the 3′ end of the 5′UTR (Fig. 2a). Several sites within the PBS domain that were protected from cleavage in the presence of HIV-2 GagΔp6 were also protected by MA but not NC, while reduced reactivities for some sequences within the 3′ end of the 5′UTR that were noted in the presence of HIV-2 GagΔp6 were also observed in the presence of NC but not MA. HIV-2 GagΔp6 or NC protected mostly purine residues, whereas mostly G residues displayed reduced cleavage in the presence of MA (Additional file 1). The protection patterns were detected in both single and double-stranded regions. Analysis of the composition of protected sites and their vicinity revealed a prevalence of G residues in the case of MA, A residues for NC, while mixed nucleotide residue composition was observed for HIV-2 GagΔp6 (Fig. 2b).

### HIV-2 MA displays high TAR annealing activity

**Fig. 3:**
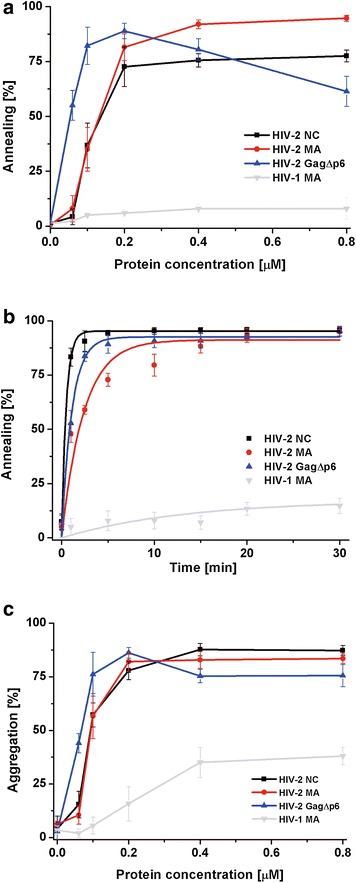
Comparison of HIV-2 GagΔp6, MA, NC and HIV-1 MA nucleic acid chaperone activity. **a** TAR annealing assays in the presence of increasing concentrations of protein. **b** Time course TAR annealing assays using 0.2 µM of each protein (1 protein per 3.9 nt). The *curves* are single-exponential fits to average data. Representative gels are included in Additional file 2. **c** TAR(−) DNA aggregation by the different proteins

The capability to facilitate the annealing of HIV-1 TAR RNA and TAR(−) DNA hairpins serves as a basic test of the in vitro NAC activity of proteins [54]. This assay resembles the annealing of (–) ssDNA to 3′UTR of viral RNA during the first strand transfer of reverse transcription. HIV-1 NC and Gag were found to be excellent nucleic acid chaperones, whereas HIV-1 MA displays little, if any, NAC activity [16, 17, 25]. We have recently shown that HIV-2 NC displays robust annealing activity of HIV-1 TAR hairpins [29], but the NAC activity of HIV-2 MA and Gag has not been reported yet. Therefore, the concentration and time course TAR RNA/TAR(−) DNA gel-shift annealing assays were performed to characterize and compare the NAC activity of HIV-2 MA, NC, and GagΔp6. A comparison of the proportion of annealed product obtained at increasing concentrations of HIV-2 MA, NC, or GagΔp6 showed that all analysed HIV-2 proteins effectively facilitated the annealing reaction (Fig. 3a). At 0.2 µM concentration (1 protein per 3.9 nt) of HIV-2 GagΔp6, NC, or MA, over 75 % of the TAR(−) DNA was annealed. However, on a molar basis, HIV-2 GagΔp6 is a more effective chaperone than NC or MA, since a twofold lower concentration of GagΔp6 was sufficient for maximal percentage of TAR annealing. Moreover, in the presence of 0.05 µM HIV-2 GagΔp6, the annealing was ~60 %, whereas no significant amount of annealed products was observed at the same concentration of HIV-2 NC or MA. Similarly to the observations made previously for HIV-1 GagΔp6 [16], we found that a further increase in HIV-2 GagΔp6 concentration led to a decrease in annealed products.

Subsequently, time course TAR annealing at 0.2 µM protein concentration (1 protein per 3.9 nt) was performed to compare the annealing rates of HIV-2 NC, MA, and GagΔp6 (Fig. 3b). We found that the annealing rate of HIV-2 NC is higher than that of GagΔp6 and MA (twofold and threefold, respectively) (Table 2). However, the final levels of TAR hairpins annealed in the presence of each analysed HIV-2 protein were similar and close to 95 %. To directly compare HIV-2 and HIV-1 MA proteins, the ability to facilitate TAR annealing was assayed for HIV-1 MA (Fig. 3a, b). In agreement with previous study [25], low level of TAR annealing (~16 %) was measured in the presence of HIV-1 MA.

**Table 2:** Annealing parameters of HIV-2 NC, MA, GagΔp6

HIV-2 protein (μM)	Annealing rate	NA annealed (%)
TAR annealing (20 mM NaCl)		
NC (0.2)	2.0 ± 0.40	95.3 ± 0.7
MA (0.2)	0.6 ± 0.05	91.1 ± 2.4
GagΔp6 (0.2)	0.9 ± 0.11	92.5 ± 1.3
tRNA^Lys3^ annealing (20 mM NaCl)		
NC (1.5)	1.87 ± 0.44	95.1 ± 1.8
MA (1.5)	0.17 ± 0.08	37.8 ± 5.0
GagΔp6 (1.5)	0.18 ± 0.06	85.1 ± 5.2
tRNA^Lys3^ annealing (150 mM NaCl)		
NC (1.5)	0.07 ± 0.03	35.8 ± 4.9
MA (1.5)	0.21 ± 0.06	48.1 ± 4.9
GagΔp6 (1.5)	0.26 ± 0.03	82.1 ± 3.4
NC (3)	0.11 ± 0.02	43 ± 5.2
MA (3)	0.22 ± 0.07	60 ± 4.9
GagΔp6 (3)	0.37 ± 0.07	90 ± 3.7
RNA dimerization (150 mM NaCl)		
NC (6)	0.48 ± 0.10	49.2 ± 1.6
GagΔp6 (6)	0.15 ± 0.01	45.7 ± 2.2

### HIV-2 MA effectively aggregates nucleic acids

The ability to sequence non-specific aggregation of NA is considered an important characteristic of NAC proteins [55]. We directly compared the NA-aggregation properties of analysed HIV-2 proteins and HIV-1 MA using sedimentation assays (Fig. 3c). In this assay, the ^32^P-labelled HIV-1 TAR(−) DNA and TAR RNA were incubated with increasing concentrations of protein, centrifuged, and the amount of non-aggregated NA was measured. We found that HIV-2 NC, MA, and GagΔp6 are effective NA-aggregating agents, since ~80 % of NA aggregation was detected at a 0.2 µM concentration of each protein. The observed NA aggregation at a given protein concentration was similar for HIV-2 NC and MA, but significantly greater for HIV-2 GagΔp6, since a two-fold lower concentration of GagΔp6 was sufficient for the maximal NA aggregation. HIV-1 MA aggregated NA much weaker than HIV-2 proteins since only up to ~40 % of aggregation was detected at the highest protein concentration used (0.8 µM).

### NC and MA domains may contribute to the tRNA^Lys3^ annealing activity of HIV-2 Gag

The HIV-1 Gag and NC, via their NAC activity, mediate tRNA^Lys3^ annealing in vitro and in vivo [14, 17, 36]. On the contrary, HIV-1 MA does not promote the tRNA^Lys3^ annealing in vitro even when high MA concentrations and long reaction times were employed [17]. The aggregation and TAR annealing assays demonstrated that HIV-2 MA displays high NAC activity in vitro (Fig. 3). To further characterize and compare the activity of the analysed HIV-2 proteins, gel-shift tRNA^Lys3^ annealing assays were performed, using the in vitro transcribed, unmodified tRNA^Lys3^, and a 560-nt RNA, corresponding to the 5′UTR of HIV-2 genomic RNA (Fig. 1b). To determine the influence of salt on the NAC activity of proteins, the annealing reactions were conducted at low (20 or 50 mM) and physiological (150 mM) NaCl concentrations.

**Fig. 4:**
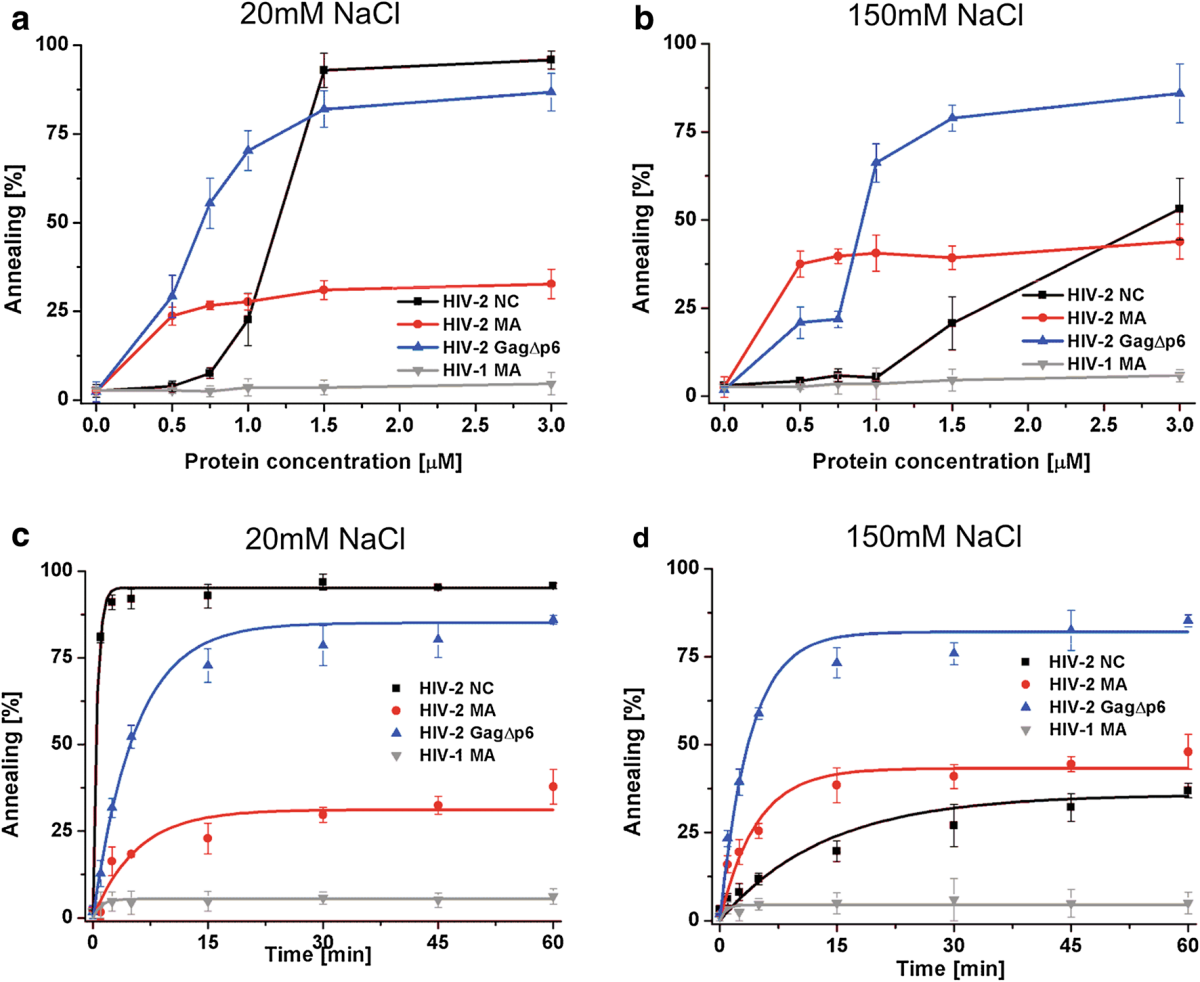
Protein induced tRNA^Lys3^ annealing in the presence of varying NaCl concentrations. **a** and **b** Concentration course annealing assays. **c** and **d** Time course annealing assays using 1.5 µM of each protein (1 protein per 3.9 nt), the *curves* are single-exponential fits to average data. Representative gels are included in Additional file 2

The annealing assays showed that HIV-2 GagΔp6 displays high tRNA^Lys3^ annealing activity and, on a molar basis, is an even better chaperone than HIV-2 NC (Fig. 4a, b). Moreover, in contrast to HIV-2 NC, GagΔp6 activity is not influenced by NaCl concentration. In the presence of 1 µM HIV-2 GagΔp6, the annealing was ~70 % at 20 and 150 mM NaCl. At the same concentration of HIV-2 NC, only 25 % of annealing was measured at 20 mM NaCl, whereas no activity was observed at 150 mM NaCl. Furthermore, at 1.5 µM concentration (1 protein per 3.9 nt) of HIV-2 NC the presence of 150 mM NaCl reduced the annealing activity by 80 % and, even at 3 µM NC (1 protein per 1.8 nt), reduction by 30 % was still observed. Interestingly, in the presence of 0.5 µM HIV-2 MA, ~25 and ~37 % annealing of tRNA^Lys3^ was achieved at 20 and 150 mM NaCl, respectively (Fig. 4a, b). These results suggest that an increase in salt concentration stimulates to some extent the activity of HIV-2 MA. Although HIV-2 MA promoted tRNA^Lys3^ annealing at lower concentrations than HIV-2 NC, in contrast to NC, a further increase in its concentration up to 3 µM (1 protein per 1.8 nt) did not lead to an increase in annealing. In our hands, and in agreement with previous results [17], HIV-1 MA did not promote tRNA^Lys3^ annealing even at high protein concentration and during an extended period of reaction (Fig. 4).

The time course tRNA^Lys3^ annealing assays performed at 1.5 µM protein concentration (1 protein per 3.9 nt), revealed important differences between the annealing rates of HIV-2 NC, MA, and GagΔp6 at 20 mM and 150 mM NaCl (Fig. 4c, d). At low NaCl, HIV-2 NC displayed a significantly higher (~11-fold) annealing rate than HIV-2 GagΔp6 or MA. The observed final percentages of tRNA^Lys3^ annealed were similar for HIV-2 NC and GagΔp6 (~95 and ~85 %, respectively), and ~38 % in the presence of HIV-2 MA. At physiological NaCl concentration, the annealing rates and final percentages of annealing in the presence of HIV-2 MA and GagΔp6 did not change significantly. Interestingly, HIV-2 NC exhibited an almost 27-fold lower annealing rate and the final percentage of tRNA^Lys3^ annealed was reduced to ~38 %. The increase in HIV-2 proteins concentration to 3 µM (1 protein per 1.8 nt) did not change the observed trend and inhibitory effect of 150 mM NaCl was still observed for HIV-2 NC but not for HIV-2 MA or GagΔp6 (Table 2).

### HIV-2 MA does not promote HIV-2 RNA dimerization

**Fig. 5:**
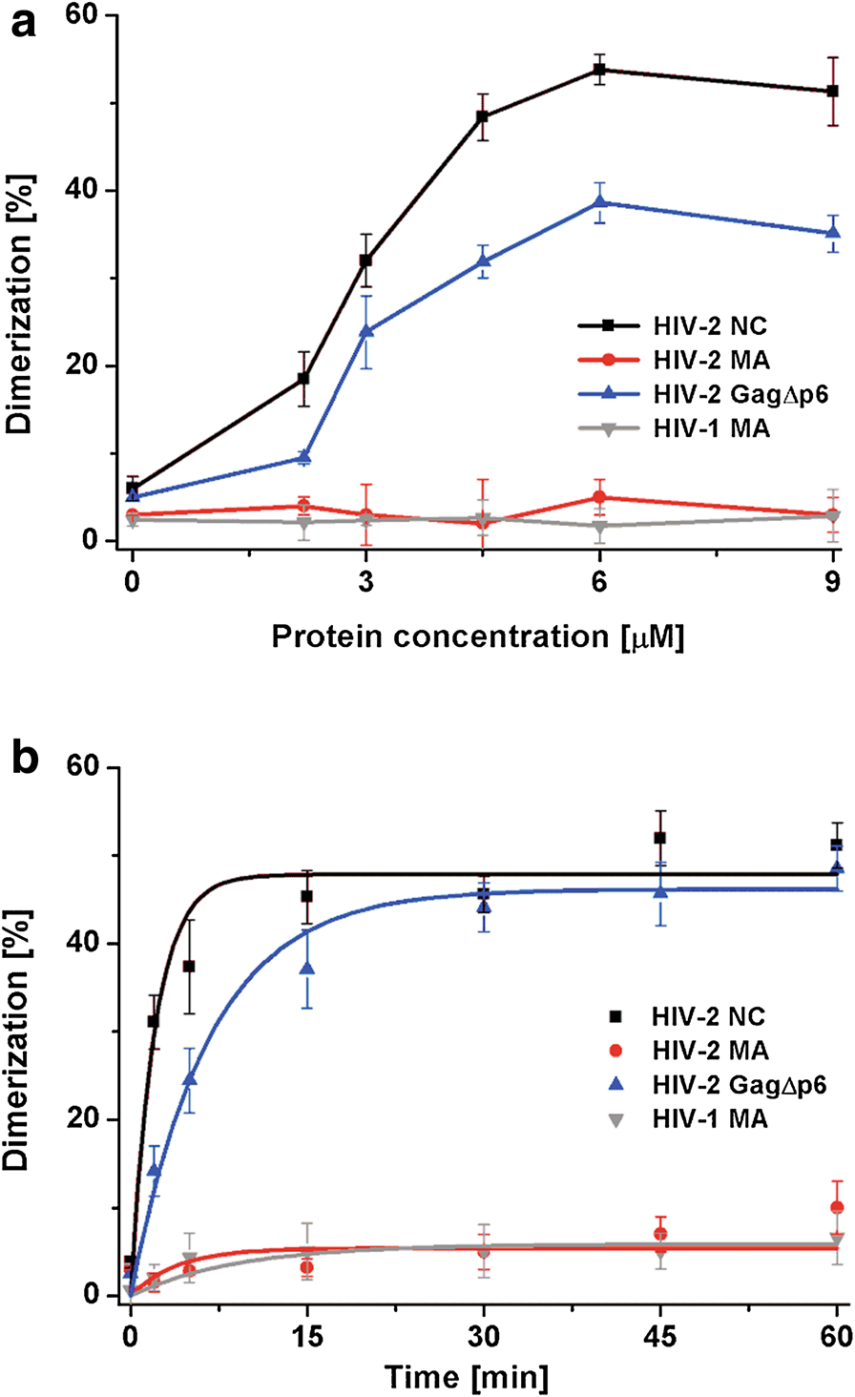
Protein-induced dimerization of HIV-2 RNA. **a** +1–444 RNA dimerization in the presence of increasing concentrations of proteins. **b** Time course dimerization in the presence of 6 µM protein (1 protein per 30 nt). The *curves* in panel **b** are single-exponential fits to average data. Representative gels are included in Additional file 2

HIV-2 genomic RNA is packaged as a dimer into the virions and, as in other retroviruses, Gag is involved in genome dimerization and packaging [35, 37, 38]. It was presented that, similarly to HIV-1, the intact NC domain within the uncleaved HIV-2 Gag confers specific binding of dimerization and Ψ signals via its two zinc finger motifs [37]. Our results showed that freestanding HIV-2 MA binds several sequences of HIV-2 RNA that are important for dimerization and packaging (Fig. 2a). To elucidate the role of the MA domain in HIV-2 RNA dimerization, in vitro assays in the presence of HIV-2 GagΔp6, MA, and NC were performed. In contrast to HIV-1, HIV-2 RNA forms tight dimers in vitro inefficiently, so the +1–444 transcript derived from the 5′UTR of HIV-2 RNA was chosen, since it forms dimers in vitro more efficiently than the entire (560 nt) 5′UTR [56]. The +1–444 transcript contains sequences proposed to be involved in HIV-2 genome dimerization: palindrome of SL1, called DIS (dimer initiation site), Ψ sequence with palindrome *pal*, and TAR domain [33, 38, 42, 56, 57]. We observed that HIV-1 MA and HIV-2 MA were unable to induce dimerization in vitro, whereas both HIV-2 NC and GagΔp6 facilitated the dimerization of +1–444 RNA (Fig. 5). In comparison to annealing assays, a significantly lower concentration of HIV-2 NC or GagΔp6 (1 protein per 30 nt) was needed to obtain the maximum percentage of RNA dimer (Fig. 5a). This observation is consistent with the hypothesis that, at the initial steps of packaging, a limited number of Gag is engaged in genome selection and dimerization [37]. Surprisingly, regardless of the presence of 150 mM NaCl in dimerization assays, HIV-2 NC was more effective on a molar basis than HIV-2 GagΔp6 and exhibited a three-fold higher dimerization rate (Fig. 5a; Table 2).

## Discussion and conclusions

In this work, we investigated the chaperone activity of HIV-2 GagΔp6, MA, and NC proteins, their binding specificity, and interactions with HIV-2 RNA. We also included HIV-1 MA in chaperone assays for direct comparison. The results of NA aggregation, TAR, or tRNA^Lys3^ annealing assays showed that, on a molar basis, HIV-2 GagΔp6 is a more robust nucleic acid chaperone than NC. Moreover, at a physiological salt concentration, the rate and final percentage of annealed tRNA^Lys3^ were significantly higher in the presence of HIV-2 GagΔp6 than HIV-2 NC (Fig. 4b, c; Table 2). The salt-dependent binding assays revealed that HIV-2 GagΔp6 binds to RNA with higher affinity than freestanding HIV-2 NC (Table 1). These observations suggest that domains other than NC contribute to the NAC activity of HIV-2 Gag. Indeed, we found that HIV-2 MA binds RNA and displays high NAC activity in vitro, since it effectively aggregated NA and facilitated the annealing of TAR hairpins (Fig. 3). This is in contrast to HIV-1 MA, which displays very poor NA aggregation and TAR annealing activity (Fig. 3) [25]. In addition HIV-1 MA does not chaperone tRNA^Lys3^ annealing in vitro while in the presence of HIV-2 MA, up to ~50 % of tRNA^Lys3^ annealing was measured (Fig. 4). In low salt concentration HIV-2 MA displays reduced tRNA^Lys3^ annealing activity compared to that of HIV-2 NC and GagΔp6. However the difference in activity is less evident in TAR annealing assays, suggesting that NAC activity of HIV-2 MA is limited by substrates length and stability to a greater degree than that of HIV-2 NC or GagΔp6.

We observed that HIV-2 GagΔp6 and MA binding to RNA is salt-independent in the range from 50 to 250 mM NaCl (Table 1). Consistently with these results, the tRNA^Lys3^ annealing activity of HIV-2 GagΔp6 and MA was not sensitive to monovalent salt at 20–150 mM (Fig. 4). Contrary to our data on HIV-2 MA, the increase in monovalent salt concentration from 50 to 150 mM significantly decreased the RNA-binding affinity of HIV-1 MA [25]. The RNA-binding properties and NAC activity of HIV-2 NC are highly salt-sensitive. At a physiological NaCl concentration, the extent and rate of tRNA^Lys3^ annealing in the presence of HIV-2 NC were lower than in the presence of HIV-2 MA (Fig. 4; Table 2). The comparison of the HIV-2 NC and MA chaperone activity at different salt concentrations supports involvement of both RNA-binding domains of HIV-2 GagΔp6 in tRNA^Lys3^ annealing.

Based on the presented results, we propose that both NC and MA domains contribute to the chaperone activity of HIV-2 Gag. Although HIV-2 NC is an effective chaperone, its activity is lower than that of HIV-1 NC [29]. Additionally to the NC domain, the MA domain via interactions with RNA and its NAC activity may enhance the activity of HIV-2 Gag. However we cannot exclude influence of other domains or multimerization on the NAC activity of HIV-2 Gag. The available data indicate that for HIV-1 the NC domain is primarily responsible for the overall NAC activity of HIV-1 Gag [16, 17], whereas the MA domain via RNA binding inhibits the NAC activity of HIV-1 Gag [17]. Interestingly, the MA domain does not influence the NAC activity of RSV Gag (alpharetrovirus) [15], while the HTLV-2 MA protein (deltaretrovirus) displays significantly higher chaperone activity than HTLV-2 NC [25]. Our data on HIV-2 indicate that the role of the MA domain in the NAC activity of Gag may differ not only between, but also within, retroviral genera.

**Fig. 6:**
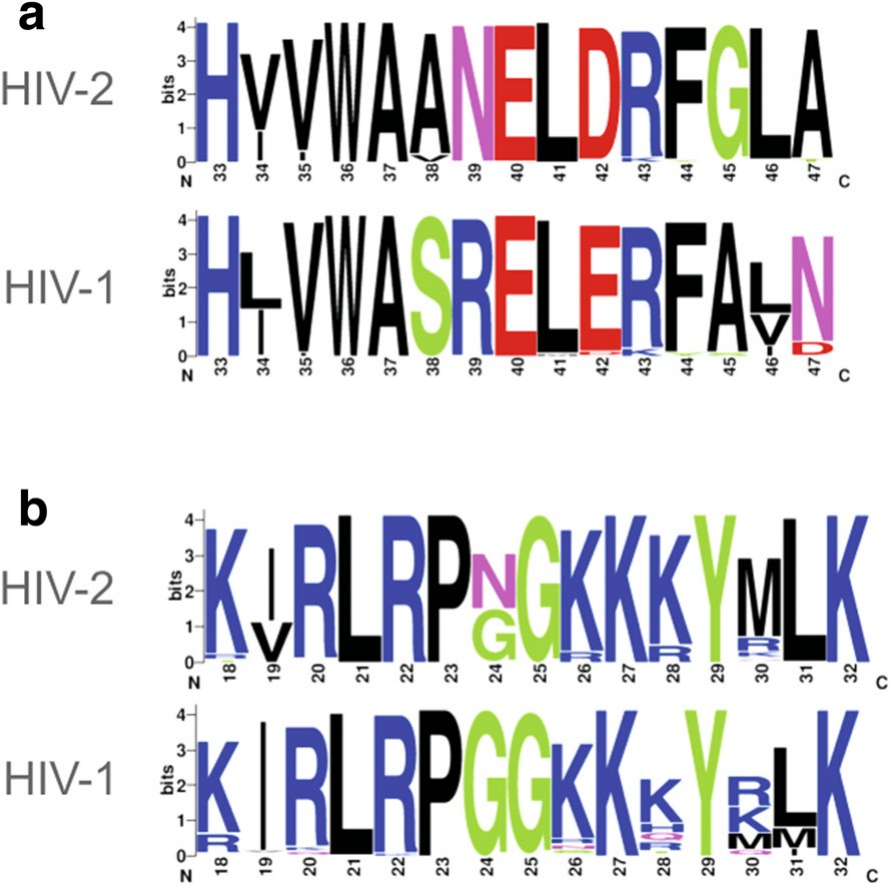
Sequence alignment of **a** MA α-helix II and **b** highly basic region (HBR); from HIV and related SIV subtypes. Sequences were taken from HIV Sequence Database (http://hiv.lanl.gov) and at least hundred sequences were align for each group. Alignments were visualized using http://weblogo.berkeley.edu/ [68]

The residues important for the chaperone activity of deltaretroviral MA proteins are highly conserved, including the presence of positively charged amino acid residues, and are located in α-helix II [25]. In case of HIV-1 MA two basic residues within the corresponding region (α-helix II) are also conserved between the HIV-1 and related SIV subtypes (Fig. 6a) but are not sufficient to confer NA chaperone activity. However, substitution of E40R/E42L/N47K in HIV-1 α-helix II resulting in the basic character mimicking HTLV-2 MA increased HIV-1 MA NAC activity to the level comparable to that of HTLV-2 [25]. In case of HIV-2 and related SIV subtypes this region is less positively charged than that of HIV-1 MA (Fig. 6a). Interestingly, calculated [58] isoelectric point is higher for the full length MA protein of HIV-2 than HIV-1 MA (9.68 and 9.10, respectively). The observed differences in the NAC activity of MA proteins may result from the more basic character of HIV-2 MA relative to HIV-1 MA. This is further supported by the analysis of electrostatic potential surfaces of HIV-1 and HIV-2 MA (Additional files 3 and 4). The amino acid residues of HBR were shown to be responsible for the RNA binding in HIV-1 MA [48]. The sequence alignment of HIV-1, HIV-2 and related SIV isolates demonstrated high level of conservation of the HBR between those retroviruses (Fig. 6b). Since HIV-1 MA do not promote tRNA^Lys3^ annealing it is unlikely that HBR region is a major determinant of HIV-2 MA chaperone activity. Analysis of the electrostatic potential surfaces of HIV-2 and HIV-1 MA proteins reveals regions other than α-helix II or HBR that might be considered important for HIV-2 MA NAC activity (Additional files 3 and 4). The difference between electrostatic potential surfaces of the two proteins was observed e.g. in the C terminus. The C terminus of MA proteins is disordered [43, 46]. Unstructured regions containing basic residues able to nonspecifically interact with nucleic acids are suggested to be important for the proteins’ NAC activity [19, 29, 47, 59]. On the other hand, relatively high salt resistance (up to 250 mM) of HIV-2 MA (Table 1) may suggest that some specific interactions contribute to HIV-2 MA/RNA binding.

A recent study has shown that HIV-1 Gag exhibits significant differences in salt-dependent binding to diverse HIV-1 RNA fragments and binds non-Ψ RNA with low specificity via its NC and MA domains, whereas binding to Ψ RNA is highly specific and only the NC domain is engaged [60]. Such a binding model is not common to all retroviral Gag polyproteins, since, in deltaretroviruses (HTLV-2), the MA domain binds RNA more specifically than NC and plays a dominant role in the initial recognition of the Ψ signal in genomic RNA [25]. Our results demonstrate that HIV-2 GagΔp6 binds both Ψ and non-Ψ RNAs with high specificity, which was manifested by a negligible change in the dissociation constants within the 50–250 mM NaCl range (Table 1). Moreover, even at 500 mM NaCl, HIV-2 GagΔp6 interacted with Ψ RNA and non-Ψ (PBS) RNA with strong affinities (Kd ≈ 125 and 314 nM, respectively). HIV-2 GagΔp6 binding to Ψ RNA was similar to that presented for HIV-1 GagΔp6, but HIV-1 GagΔp6 did not bind non-Ψ RNA (TARpA) at 500 mM NaCl [60]. HIV-2 GagΔp6 bound TARpA at 500 mM NaCl but with affinity (Kd ≈ 965 nM) lower than PBS. At low ionic strength (50 mM NaCl), HIV-2 NC binding to Ψ RNA was comparable to that of HIV-2 GagΔp6, but highly susceptible to salt concentration. HIV-2 NC and MA bound non-Ψ RNA with comparable affinity, but MA binding to Ψ RNA was notably weaker than that of NC or GagΔp6. Taken together, our results suggest contributions of both the NC and MA domains to the interactions of HIV-2 Gag with RNA, but the NC domain plays a major role in recognizing the Ψ signal. Our results showing that HIV-2 GagΔp6 and NC, but not MA, occupy some of the sites within the Ψ region of 5′UTR may further support this notion. Although the majority of HIV-2 GagΔp6, NC, and MA binding sites cluster within the Ψ region of HIV-2 5′UTR, extensive interactions were also detected within the TAR and PBS domains (Fig. 2). A recent study demonstrated that the RNA-binding specificity of HIV-1 Gag changes during viral replication [31]. Only a few regions of the viral RNA interacted with HIV-1 Gag in cytosol, including the 5′UTR, while the entire RNA was covered within the virus particles. Interestingly, the binding of HIV-1 Gag to TAR observed in the cytosol was HIV-1 subtype-dependent. Several lines of evidence suggested that TAR might be important for HIV dimerization and packaging [61, 62]. Moreover, TAR stability is considered important in the strand transfer during reverse transcription [63] and may also influence the Gag translation efficiency [64]. Our in vitro conditions are likely in favour of the detection of the high-affinity binding sites, but without differentiation of the replication stage. Binding of HIV-2 GagΔp6, NC, and MA to PBS in the vicinity of the tRNA^Lys3^ binding regions may support their involvement in primer annealing. Indeed, all proteins promoted tRNA^Lys3^ annealing to the 5′UTR (Fig. 4). Interestingly, findings that HIV-1 Gag has a strong preference for G-rich binding sites in cells and A-rich in virions [31] is reflected in our in vitro binding studies, showing a high purine content within the HIV-2 GagΔp6 binding sites.

In HIV-2 RNA, packaging and dimerization signals overlap, and the NC domain of HIV-2 Gag is proposed to be required for viral genome dimerization and packaging in vivo [37, 38, 56]. Indeed, we found that HIV-2 NC and GagΔp6 effectively promote in vitro dimerization of HIV-2 RNA containing DIS and *pal* dimerization signals (Fig. 5). For HIV-1 Gag, numerous lines of evidence support the involvement of the NC domain in the genomic RNA selection, dimerization, and packaging, but several observations suggest a contribution of the MA domain to these processes [41]. It was found that the presence of either the NC or the MA domain is required for genome packaging during HIV-1 particle assembly [23, 40]. However, a recent study revealed that the MA domain of HIV-1 Gag binds almost exclusively to specific cellular tRNAs [31]. Our data suggest that the MA domain is dispensable for HIV-2 GagΔp6-promoted dimerization, since this process is not supported by HIV-2 MA in vitro. On the other hand, HIV-2 MA binds some *cis*-acting dimerization and packaging sequences in 5′UTR of HIV-2 RNA (Fig. 2). Therefore, we cannot exclude the participation of the MA domain in HIV-2 genome selection and dimerization in the cell.

## Methods

### Cloning, expression and purification of recombinant proteins

The HIV-2_ROD_ NC protein was obtained using pGEX-4T-3-NCp8 as described previously [33]. Sequences encoding HIV-2 MA, GagΔp6 (HIV-2_ROD_ isolate) and HIV-1 MA (HIV-1_NL4-3_ isolate) were PCR amplified from HIV-2 pROD10-EVA232 and pNL4-3-ARP2006 (National Institute for Biological Standards and Control, Centre for AIDS Reagents, UK). PCR products were digested, purified using PureLink^®^ spin columns (Invitrogen), and cloned into a pGEX-4T-3 expression vector. The sequence of each construct was confirmed by DNA sequencing. The glutathione S-transferase (GST) fusion HIV-2 NC, MA, and GagΔp6 and HIV-1 MA recombinant proteins were expressed in One Shot^®^ BL21(DE3)pLysS *E. coli* (Invitrogen) and purified by affinity chromatography on Glutathione Sepharose (GE Healthcare) as described in the Additional file 5. The GST tag was removed by thrombin cleavage. The purity of proteins was assessed by SDS–PAGE and estimated to be above 90 %. Protein concentrations were determined by their absorption spectrum and protein samples were aliquoted and stored at −80 °C.

### DNA and RNA substrates

TAR(−) DNA, corresponding to the trans activation response (TAR) sequences of HIV-1_MAL_, was ^32^P-labelled at the 5′-end with [γ-^32^P]ATP using T4 polynucleotide kinase (Fermentas) and purified using NucAway Spin Columns (Life Technologies). HIV-1 TAR RNA and unmodified human tRNA^Lys3^ (referred to here as tRNA^Lys3^) were obtained using a PCR-generated template (Additional file 6) and Ambion T7-MEGAshortscript. Transcripts were purified by denaturing gel electrophoresis (8 M urea) in 1 × TBE, followed by elution and ethanol precipitation. The tRNA^Lys3^ was 3′-end labelled using [α-^32^P]pCp and T4 RNA ligase (Fermentas) and purified on G50 columns (GE Healthcare). Templates for in vitro transcription of HIV-2 RNA molecules were obtained by PCR amplification of fragments from the HIV-2 plasmid pROD10-EVA232 using a forward primer containing a T7 promoter sequence (Additional file 6). The RNA molecules were as follows: TARpA (nt +1–188), PBS (nt +197–379), Ψ (nt +380–560), RNA +1–444, 5′UTR (nt +1–560) and RNA +1–891. RNAs were synthesized using T7-MEGAscript (Ambion) and purified using Direct-zol™ RNA MiniPrep (Zymo Research). The integrity of the RNAs was assessed by agarose gel electrophoresis under denaturing conditions. Purified RNA was stored at −20 °C. For some assays, RNA was 3′-end labelled using [α-^32^P]pCp and T4 RNA ligase (Fermentas) following purification using Direct-zol™ RNA MiniPrep (Zymo Research).

### Filter-binding assay

Equilibrium-binding experiments were performed as described previously [59] with the following modifications. Reactions were carried out in binding buffer (20 mM HEPES–KOH pH 7.5, 1 mM MgCl_2_, 10 µM TCEP, 5 mM β-mercaptoethanol, 10 µM ZnCl_2_, and 50–500 mM NaCl). The final concentration of RNA was 0.2 nM. The binding reactions were incubated for 25 min at room temperature and then 50 µl of each reaction was filtered and washed with 200 µl of binding buffer containing 50 mM NaCl. After filtration, the membranes were dried and exposed to a phosphoimager screen. Data were analysed using Multigauge (Fuji) and Origin (OriginLab) software.

### Hydroxyl radical footprinting and detection of RNA cleavage products

RNA +1–891 was used for footprinting experiments (Additional file 6) and the secondary structure of the 5′UTR within this RNA was confirmed previously [33]. RNA samples (5 pmol) were heated at 95 °C for 1 min and slowly cooled to 4 °C. Subsequently, buffer was added to the final concentration of 40 mM Tris–HCl, pH 8.0, 130 mM KCl, 0.5 mM EDTA, and 5 mM MgCl_2_, and samples were incubated for 25 min at 37 °C. Folded RNA samples were diluted 20-fold with 20 mM Tris–HCl, pH 8.0, followed by addition of NC, MA, or GagΔp6 (6 μl of 3 µM, 6 µM, or 12 µM protein in the buffer containing 50 mM Tris–HCl, pH 8.0, 1 M NaCl, 6.7 mM β-mercaptoethanol, 2.5 mM DTT, 0.1 mM ZnCl_2_) to a 70 μl reaction. RNA/protein complexes were formed at 0 °C for 20 min. Footprinting reactions were initiated by applying on the wall of the tube 1 μl of 2.5 mM (NH_4_)Fe(SO_4_)_2_, 50 mM sodium ascorbate, 1.5 % H_2_O_2_, and 2.75 mM EDTA, and centrifugating. After 15 s at 24 °C, reactions were quenched by the addition of 20 μl of stop solution containing 0.1 M thiourea and 0.2 M EDTA. RNA were purified using Direct-zol RNA MiniPrep Kit (Zymo Research). For the reverse transcription reactions, a total of 1.5 pmols of RNA was mixed with 2 µl of fluorescently labelled primer 186, 540, or 787 (Additional file 6) [4 μM Cy5 (with reagent) and 6 uM Cy5.5 (without reagent)] and 12 μl of primer-template solutions were incubated at 85 °C for 3 min, 60 °C for 5 min, 35 °C for 5 min, and 50 °C for 2 min. Reverse transcription and sample processing were carried out as previously described [65]. Sequencing ladders were prepared using a Thermo Sequenase Cycle Sequencing Kit (Affymetrix) according to the manufacturer’s protocol. Samples and sequencing ladders were purified using a ZR DNA Sequencing Clean-up Kit (Zymo Research) and analysed on a GenomeLab GeXP Analysis System (Beckman-Coulter). Three to nine repetitions were obtained for each read. Electropherogram peaks were converted to reactivity values using Shapefinder software [66]. Reverse transcription stops in the control reaction were identified as outlying high peaks in the plotted background area. To normalize the data, peak intensity for each nucleotide was divided by the average intensity of the 8 % most reactive peaks excluding outliers. The outliers were defined as greater than 1.5 times the interquartile difference above the 3rd quartile [67]. Normalized data were averaged and nucleotide positions corresponding to reverse transcription stops were excluded from further analysis. Differences between reactivity values for reaction without protein and containing protein were calculated. The consistent drop in reactivity with increasing protein concentration larger than at least 20 % of reactivity value was regarded as a possible binding site.

To estimate number of binding sites within different domains of 5′UTR for HIV-2 GagΔp6, NC and MA, number of residues protected from hydroxyl radical cleavage in the presence of only GagΔp6 and NC were compared to the number of those protected only in the presence of GagΔp6 and MA.

### TAR annealing assay

^32^P-labelled HIV-1 TAR(−) DNA (1 nM) and unlabelled HIV-1 TAR RNA (6 nM) were heat denaturated and folded separately in buffer containing 20 mM Tris–HCl, pH 7.5, 30 mM NaCl, 0.1 mM MgCl_2_, 10 µM ZnCl_2_, and 5 mM DTT. Then, the mixture of both oligonucleotides was incubated with increasing concentrations of each protein (0–0.8 µM) at 37 °C for 5 min. The time course assays were conducted at 37 °C using 0.2 µM protein and the samples were removed at the indicated time points. All reactions were quenched with 0.5 volume of stop solution (20 % glycerol, 20 mM EDTA pH 8.0, 0.1 % SDS, 0.25 % bromophenol blue, and 0.4 mg/ml yeast tRNA). Samples were analysed by native PAGE (8 %) in 0.5 × TBE at 4 °C.

### Sedimentation assays

^32^P-labelled HIV-1 TAR(−) DNA (1 nM) was combined with complementary unlabelled TAR RNA in a buffer containing 50 mM Tris pH 7.5, 20 mM NaCl, and 0.2 mM MgCl_2_. Reactions (10 µl) were incubated with increasing protein concentrations (0–0.8 µM) at 37 °C for 5 min. Subsequently, the mixtures were centrifuged at 11,400 rpm for 20 min. Supernatants (2 µl) were collected and subjected to scintillation counting.

### tRNA^Lys3^ annealing assay

^32^P-labelled tRNA^Lys3^ (2 nM) and unlabelled +1–560 HIV-2 RNA (10 nM) were refolded in 50 mM Tris–HCl, pH 7.5 by heating at 95 °C for 1 min and slow cooling to 37 °C, followed by addition of MgCl_2_ to 10 mM and placement on ice. The annealing buffer contained 50 mM Tris–HCl pH 7.5, 5 mM DTT, and 1 mM MgCl_2_, and different NaCl concentrations of 20, 50, or 150 mM. The mixture was incubated at 37 °C for 10 min, followed by addition of protein and further incubation for 10 min. The time course assays were conducted at 37 °C using 1.5 or 3 µM protein and the aliquots were removed at the indicated time points. All reactions were quenched by incubation with 1 % (w/v) SDS at room temperature for 5 min. The samples were phenol/chloroform-extracted, mixed with loading buffer (50 % glycerol with dyes), and separated on 1.4 % SDS-agarose gel in 1 × TBE at room temperature.

### RNA dimerization

The unlabelled +1–444 HIV-2 (400 nM) spiked with the trace amount of the same ^32^P-labelled transcript was heat denatured in 50 mM Tris–HCl pH 7.5, 40 mM KCl, 150 mM NaCl, and 0.1 mM MgCl_2_. The mixture was slowly cooled to 37 °C and placed on ice, followed by addition of different concentrations of proteins. The dimerization was allowed to proceed at 37 °C for 30 min. The time course dimerization assays were conducted at 37 °C using 6 µM protein and the aliquots were removed at the indicated time points. All dimerization reactions were quenched by incubation with 1 % (w/v) SDS at room temperature for 5 min, phenol/chloroform-extracted, and mixed with loading buffer (50 % glycerol with dyes). The products were separated on 1 % agarose gel in 1 × TBE at room temperature.

All gels were autoradiographed and quantitatively analysed by phosphorimaging using a FLA-5100 phosphorimager with MultiGaugeV 3.0 software (FujiFilm). The obtained data were analysed using Origin (OriginLab) software. All graphs represent averaged data from three or more independent experiments with standard deviations indicated. In all cases, at least three independent experiments were performed, and the data presented are representative of the whole.

## Additional files

10.1186/s12977-016-0245-1 Analysis of the nucleotides identity at the HIV-2 GagΔp6, NC and MA binding sites within HIV-2 5′UTR RNA. 
10.1186/s12977-016-0245-1 The representative electrophoretic analyses of the TAR RNA/TAR(−) DNA and tRNALys3/HIV-2 5′UTR annealing assays, and +1–444 RNA dimerization assays in the presence of HIV-2 GagΔp6, NC, MA and HIV-1 MA.
10.1186/s12977-016-0245-1 MA proteins of HIV-1 (PDB id. 2HMX) and HIV-2 (PDB id. 2K4E) represented by their molecular surfaces.
10.1186/s12977-016-0245-1 Movie file. 3D view of MA proteins of HIV-1 (PDB id. 2HMX) and HIV-2 (PDB id. 2K4E) represented by their molecular surfaces. 
10.1186/s12977-016-0245-1 Detailed description of HIV-2 NC, MA, GagΔp6 and HIV-1 MA proteins expression and purification. 
10.1186/s12977-016-0245-1 Table containing sequences of the primers used in this study.
