# Supplementary material for: The matrix domain contributes to the nucleic acid chaperone activity of HIV-2 Gag
Source: Retrovirology. 2016 Mar 17;13:18. doi: 10.1186/s12977-016-0245-1 (PMC4794849; doi:10.1186/s12977-016-0245-1)
Supplement: Supplementary file 1 — 10.1186/s12977-016-0245-1 Analysis of the nucleotides identity at the HIV-2 GagΔp6, NC and MA binding sites within HIV-2 5′UTR RNA. [file 12977_2016_245_MOESM1_ESM.pdf]

Additional file 1

The matrix domain contributes to the nucleic acid chaperone activity of HIV-2 Gag

Katarzyna Pachulska-Wieczorek, Leszek Błaszczyk, Marcin Biesiada, Ryszard W. Adamiak and Katarzyna J. Purzycka

Analysis of the nucleotides identity at the HIV-2 GagΔp6, NC and MA binding sites within HIV-2 5'UTR RNA. Nucleotides displaying strongest decrease of hydroxyl radical cleavage in the presence of each protein and occurring within the site where at least two consecutive residues showed decrease were counted.

Protein\residue	A	C	G	U
GagΔp6	41	16	46	15
NC	33	16	28	13
MA	16	17	27	8
