# Supplementary material for: The matrix domain contributes to the nucleic acid chaperone activity of HIV-2 Gag
Source: Retrovirology. 2016 Mar 17;13:18. doi: 10.1186/s12977-016-0245-1 (PMC4794849; doi:10.1186/s12977-016-0245-1)

Additional file 2

The matrix domain contributes to the nucleic acid chaperone activity of HIV-2 Gag

Katarzyna Pachulska-Wieczorek, Leszek Błaszczyk, Marcin Biesiada, Ryszard W. Adamiak and Katarzyna J. Purzycka

The representative electrophoretic analysis of the concentration course TAR RNA / TAR (–)DNA annealing assays. Lanes 0 contain a control sample lacking protein. The protein concentrations are indicated above each lane.

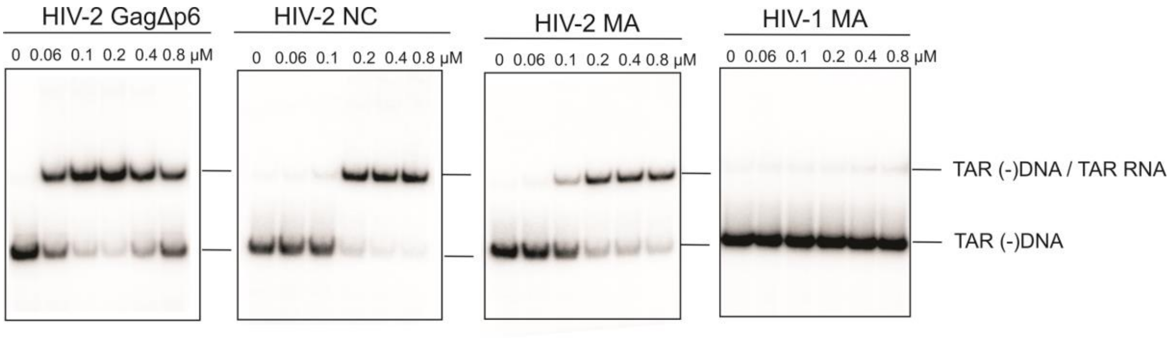

The representative electrophoretic analysis of the time course TAR RNA / TAR (–)DNA annealing assays. Reaction times are indicated above each lane.

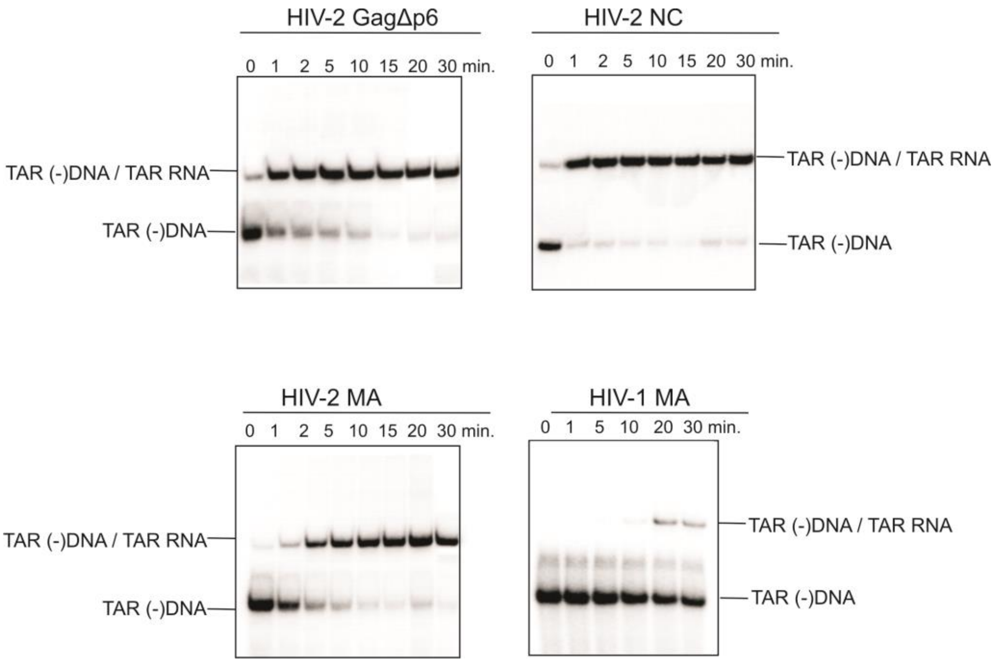

The representative electrophoretic analysis of the concentration course $tRNA^{Lys3}$ annealing assays. Lanes 0 contain a control sample lacking protein. The protein concentrations are indicated above each lane.

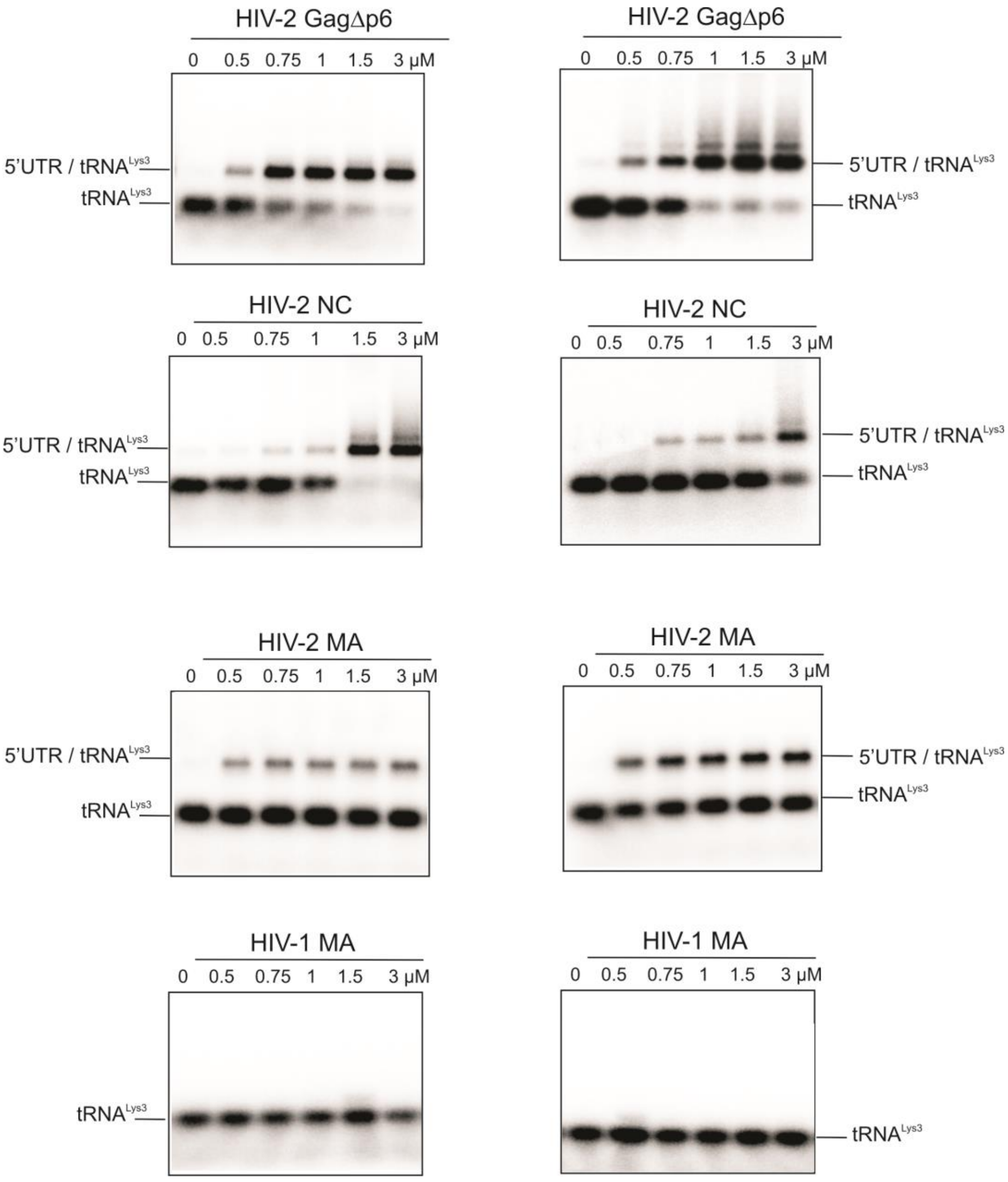

The representative electrophoretic analysis of the time course tRNA^{Lys3} annealing assays. Reaction times are indicated above each lane.

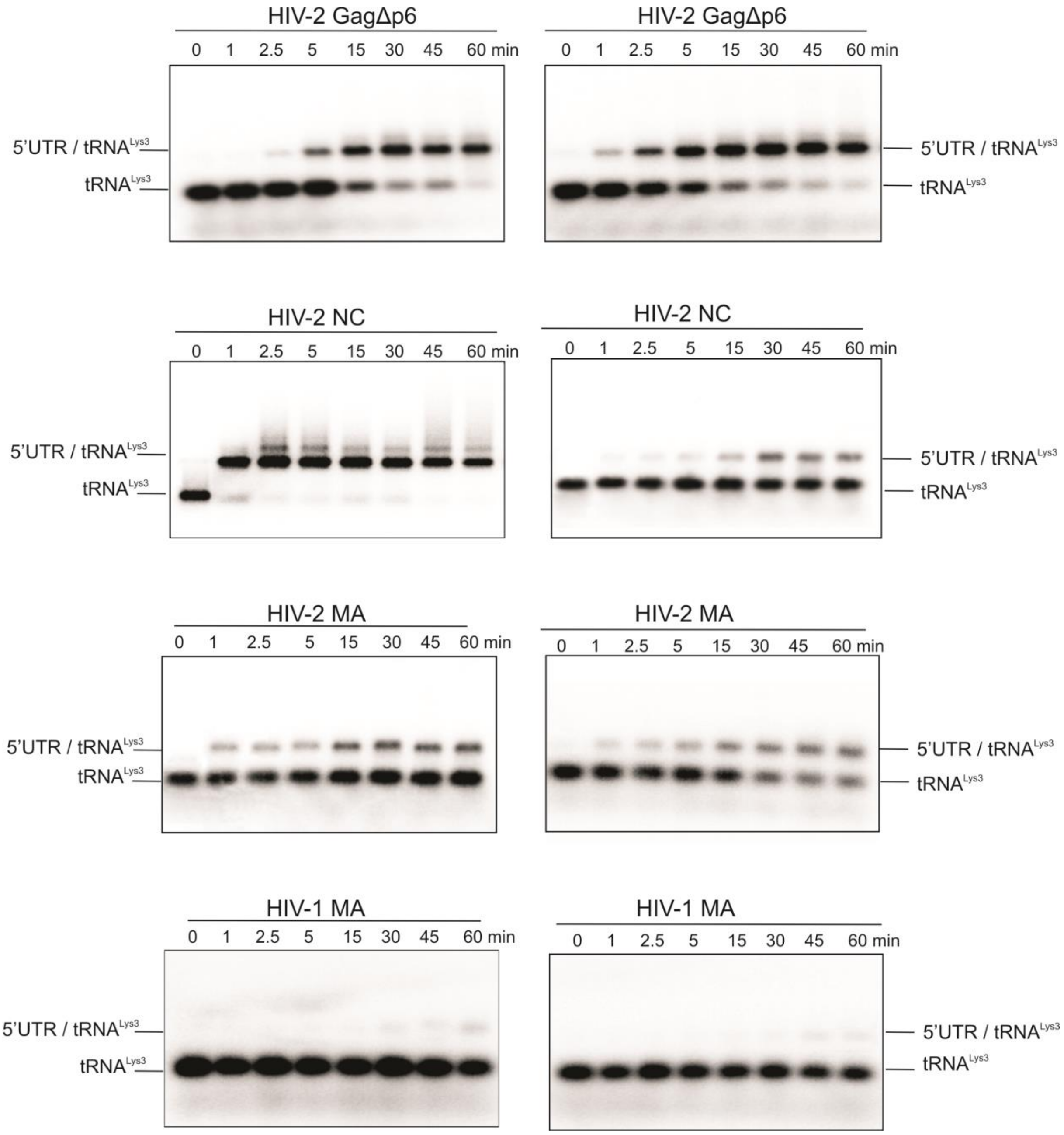

The representative electrophoretic analysis of the concentration course HIV-2 RNA dimerization assays. Lanes 0 contain a control sample lacking protein. The protein concentrations are indicated above each lane. The monomer (M) and dimer (D) bands of HIV-2 +1 – 444 RNA are indicated.

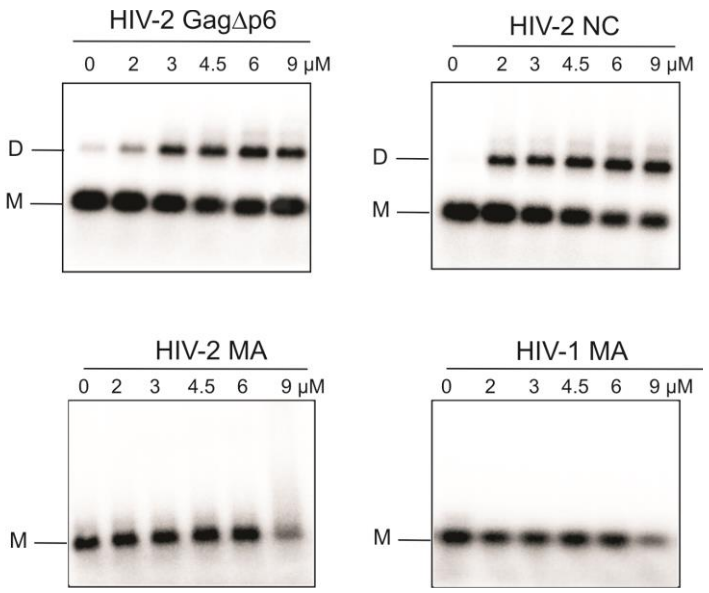

The representative electrophoretic analysis of the time course HIV-2 RNA dimerization assays. Reaction times are indicated above each lane. The monomer (M) and dimer (D) bands of HIV-2 +1 – 444 RNA are indicated.

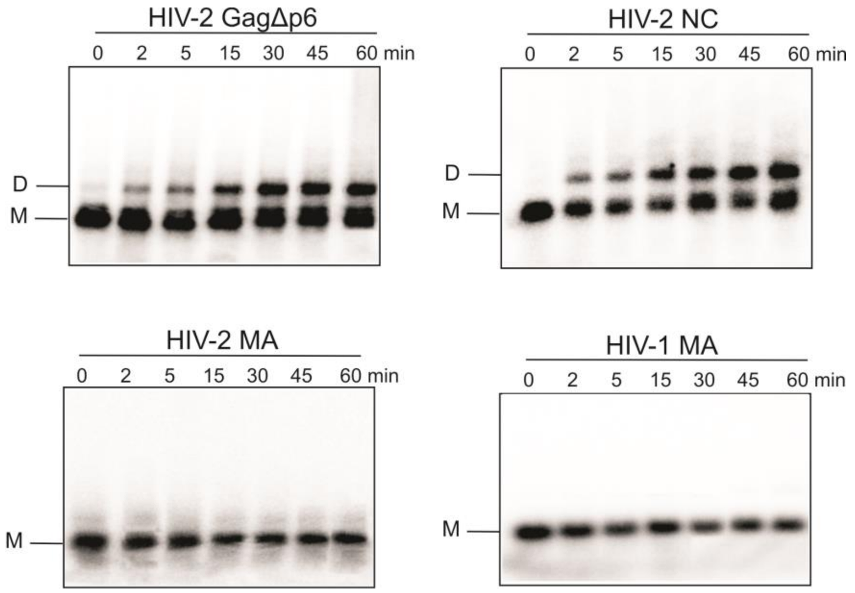

Supplement: Supplementary file 2 — 10.1186/s12977-016-0245-1 The representative electrophoretic analyses of the TAR RNA/TAR(−) DNA and tRNALys3/HIV-2 5′UTR annealing assays, and +1–444 RNA dimerization assays in the presence of HIV-2 GagΔp6, NC, MA and HIV-1 MA. [file 12977_2016_245_MOESM2_ESM.pdf]
