# Supplementary material for: The matrix domain contributes to the nucleic acid chaperone activity of HIV-2 Gag
Source: Retrovirology. 2016 Mar 17;13:18. doi: 10.1186/s12977-016-0245-1 (PMC4794849; doi:10.1186/s12977-016-0245-1)

# Additional file 3

## The matrix domain contributes to the nucleic acid chaperone activity of HIV-2 Gag

Katarzyna Pachulska-Wieczorek, Leszek Błaszczyk, Marcin Biesiada, Ryszard W. Adamiak and Katarzyna J. Purzycka

MA proteins of HIV-1 (PDB id. 2HMX) **(A)** and HIV-2 (PDB id. 2K4E) **(B)** represented by their molecular surfaces. A green arrow denotes the N terminus. Electrostatic potentials were calculated using Adaptive Poisson-Boltzmann Solver (APBS) [1] and visualized using PyMOL. Additional movie file (Additional File 4) presents this in more detail.

[1] Baker NA, Sept D, Joseph S, Holst MJ, McCammon JA. Electrostatics of nanosystems: application to microtubules and the ribosome. Proc. Natl. Acad. Sci. USA 2001;98(18): 10037-10041. doi: 10.1073/pnas.181342398

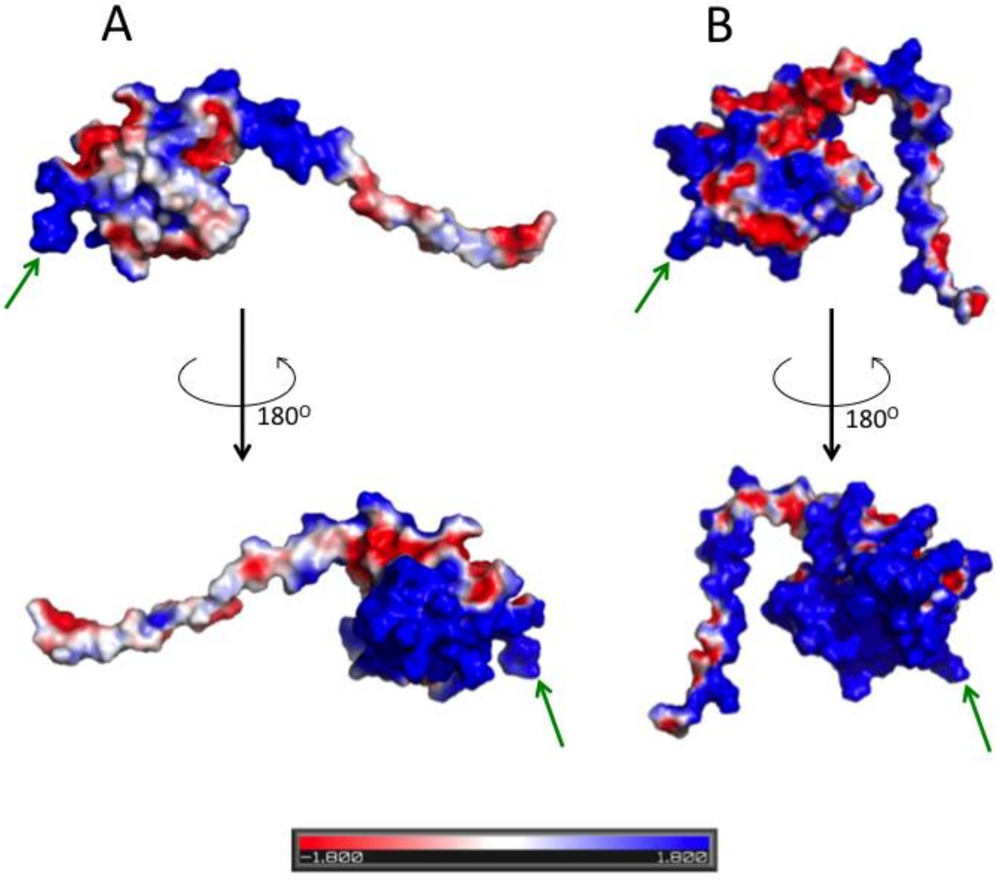

Supplement: Supplementary file 3 — 10.1186/s12977-016-0245-1 MA proteins of HIV-1 (PDB id. 2HMX) and HIV-2 (PDB id. 2K4E) represented by their molecular surfaces. [file 12977_2016_245_MOESM3_ESM.pdf]
