# Supplementary material for: The matrix domain contributes to the nucleic acid chaperone activity of HIV-2 Gag
Source: Retrovirology. 2016 Mar 17;13:18. doi: 10.1186/s12977-016-0245-1 (PMC4794849; doi:10.1186/s12977-016-0245-1)
Supplement: Supplementary file 5 — 10.1186/s12977-016-0245-1 Detailed description of HIV-2 NC, MA, GagΔp6 and HIV-1 MA proteins expression and purification. [file 12977_2016_245_MOESM5_ESM.pdf]

Additional file 5

The matrix domain contributes to the nucleic acid chaperone activity of HIV-2 Gag

Katarzyna Pachulska-Wieczorek, Leszek Błaszczyk, Marcin Biesiada, Ryszard W. Adamiak and Katarzyna J. Purzycka

Protein expression and purification

The GST fusion HIV-2 NC, MA, GagΔp6 and HIV-1 MA recombinant proteins were expressed in One Shot® BL21(DE3)pLysS Chemically Competent *E. coli* cells (Invitrogen). *E. coli* cells were grown in the LB medium with ampicilin (50 µg/ml) and chloramphenicol (34 µg/ml) at 37°C (NC and MA) or 28°C (GagΔp6) to an OD_{600} of 0.7 after which protein expression was induced with 0.8 mM IPTG. The cells were pelleted 3.5 h after incubation by centrifugation at 4000 rpm for 10 min and frozen at -80°C. The cells were resuspended in 50 ml of lysis buffer: for NC and MA proteins (50 mM Tris-HCl pH 7.5, 300 mM KCl, 1% Triton X-100, 0.5 mg/ml lysozyme, protein inhibitor cocktail and 50 or 5 mM DTT for NC and MA respectively), for GagΔp6 (50 mM Tris-HCl pH 8.0, 1M NaCl, 10 mM β-mercaptoethanol, 2.5 mM DTT, 0.1 mM $ZnCl_2$, 0.5 mg/ml lysozyme and protein inhibitor cocktail). After incubation on ice for 30 min, the lysate was sonicated 25 x 2 seconds on ice. The cell debris was pelleted by centrifugation at 20000 g for 25 min at 4°C. The nucleic acids were precipitated with 0.45% polyethyleneimine and debris was pelleted by centrifugation at 25000 g for 30 min at 4°C. The supernatant was mixed with 1 ml of Glutathione Sepharose 4B and incubated for 1h at 4°C with gentle agitation following centrifugation at 700 g for 5 min. For NC and MA proteins the Glutathione Sepharose beads were loaded onto the column and washed repeatedly with buffer I (PBS pH 7.4, 1% Triton X-100 and 50 or 5 mM DTT for NC and MA respectively), buffer II (50 mM Tris-HCl pH 7.5, 300 mM NaCl and 50 or 5 mM DTT for NC and MA respectively), buffer I and buffer III (50 mM Tris-HCl pH 7.5, 100 mM KCl, 10% glycerol and 50 or 5 mM DTT for NC and MA respectively). GagΔp6 containing Glutathione Sepharose beads were washed with purification buffer (50 mM Tris-HCl pH 8.0, 1M NaCl, 10 mM β-mercaptoethanol, 2.5 mM DTT, 0.1 mM $ZnCl_2$). GST tag was removed by thrombin cleavage (GE Healthcare) at 4°C for 12 h with gentle agitation. Thrombin cleavage was carried out in PBS buffer pH 7.4 (NC and MA) or purification buffer (GagΔp6). Protein samples were exchanged into storage buffer: HIV-2 NC (30 mM HEPES-KOH pH 6.5,

30 mM NaCl and 0.1 mM $ZnCl_2$), HIV-1 and HIV-2 MA (50 mM Tris-HCl pH 8.0, 200 mM NaCl, 1 mM DTT and 5 mM β-mercaptoethanol). HIV-2 GagΔp6 was kept in the purification buffer.
