# Supplementary material for: The matrix domain contributes to the nucleic acid chaperone activity of HIV-2 Gag
Source: Retrovirology. 2016 Mar 17;13:18. doi: 10.1186/s12977-016-0245-1 (PMC4794849; doi:10.1186/s12977-016-0245-1)
Supplement: Supplementary file 6 — 10.1186/s12977-016-0245-1 Table containing sequences of the primers used in this study. [file 12977_2016_245_MOESM6_ESM.pdf]

Additional file 6

The matrix domain contributes to the nucleic acid chaperone activity of HIV-2 Gag

Katarzyna Pachulska-Wieczorek, Leszek Błaszczyk, Marcin Biesiada, Ryszard W. Adamiak and Katarzyna J. Purzycka

Primers used in this study.

Molecule	Sequence
Generation of templates for *in vitro* transcription	
HIV-1 TAR	5'-*TAATACGACTCACTATA*GGTCTCTCTTGTTAGACCAGG-3'<br>5'-GGGTTCCTTGCTAGCCAGAGAGCTCCCGGGCTCGACCTGGTCTAACAAGAGAGACC-3'
tRNA^{Lys3}	5'-*TAATACGACTCACTATA*GCCCGGATAGCTCAGTCGGTAGAGCATCAGACTTTTAATCTGAGGGTCCAG-3'<br>5'-TGGCGCCCGAACAGGGACTTGAACCCTGGACCCTCAGATT-3'
HIV-2 TARpA	5'-*GAATTCTAATACGACTCACTATA*GGGTCGCTCTGCGGAGAG-3'<br>5'-CACACACTTAACTTGCTTCT-3'
HIV-2 PBS	5'-*GAATTCTAATACGACTCACTATA*GTCCTAGTCGCCGCCT-3'<br>5'-TCCTGCCGCCCTTACTGCCT-3'
HIV-2 Ψ	5'-*GAATTCTAATACGACTCACTATA*GACAAACCACGACGGAGTGCT-3'<br>5'-GTTTCTCGCGCCCATCTCCCACAATCTTCTACCTG-3'
+1 – 444 HIV-2 RNA	5'-*GAATTCTAATACGACTCACTATA*GGGTCGCTCTGCGGAGAG-3'<br>5'-CGCTCCACACGCTGCCTTTG-3'
+1 – 560 HIV-2 RNA	5'-*GAATTCTAATACGACTCACTATA*GGGTCGCTCTGCGGAGAG-3'<br>5'-GTTTCTCGCGCCCATCTCCCACAATCTTCTACCTG-3'
+1 – 891 HIV-2 RNA	5'-*GAATTCTAATACGACTCACTATA*GGGTCGCTCTGCGGAGAG-3'<br>5'-CTGCAGTTCCTGTTTCTGCCACTA-3'
Reverse transcription	
186	5'-CGACTAGGAGAGATGGGAGC-3'
540	5'-GTTTCTCGCGCCCATCTCCCA-3'
787	5'-GCACCAAATGACGCAGACAG-3'

[a]Promotor of T7 polymerase is annotated in italic.
